# Evaluating Work Impairment as a Source of Financial Toxicity in Cancer Healthcare and Negative Impacts on Health Status

**DOI:** 10.1158/2767-9764.CRC-23-0038

**Published:** 2023-07-05

**Authors:** Dinesh Pal Mudaranthakam, Nicole Nollen, Jo Wick, Dorothy Hughes, Danny Welch, Elizabeth Calhoun

**Affiliations:** 1 Department of Biostatistics & Data Science, University of Kansas Medical Center, Kansas City, Kansas.; 2 Department of Population Health, University of Kansas Medical Center, Kansas City, Kansas.; 3 University of Kansas Comprehensive Cancer Center, Kansas City, Kansas.; 4 Population Health Sciences, University of Illinois Chicago, Chicago, Illinois.

## Abstract

How the socioeconomic factors intersect for a particular patient can determine their susceptibility to financial toxicity, what costs they will encounter during treatment, the type and quality of their care, and the potential work impairments they face. The primary goal of this study was to evaluate financial factors leading to worsening health outcomes by the cancer subtype. A logistic model predicting worsening health outcomes while assessing the most influential economic factors was constructed by the University of Michigan Health and Retirement Study. A forward stepwise regression procedure was implemented to identify the social risk factors that impact health status. Stepwise regression was done on data subsets based on the cancer types of lung, breast, prostate, and colon cancer to determine whether significant predictors of worsening health status were different or the same across cancer types. Independent covariate analysis was also conducted to cross-validate our model. On the basis of the model fit statistics, the two-factor model has the best fit, that is, the lowest AIC among potential models of 3270.56, percent concordance of 64.7, and a C-statistics of 0.65. The two-factor model used work impairment and out-of-pocket costs, significantly contributing to worsening health outcomes. Covariate analysis demonstrated that younger patients with cancer experienced more financial burdens leading to worsening health outcomes than elderly patients aged 65 years and above. Work impairment and high out-of-pocket costs were significantly associated with worsening health outcomes among cancer patients. Matching the participants who need the most financial help with appropriate resources is essential to mitigate the financial burden.

**Significance:**

Among patients with cancer, work impairment and out-of-pocket are the two primary factors contributing to adverse health outcomes. Women, African American or other races, the Hispanic population, and younger individuals have encountered higher work impairment and out-of-pocket costs due to cancer than their counterparts.

## Introduction

Cancer is an incredibly pervasive and complex set of diseases, with 18.1 million new cancer cases and 9.5 million cancer-related deaths worldwide in 2018 (1). On the basis of recent incidence rates, mortality rates, and estimated number of cases, the most common cancers are breast, lung, prostate, and colon cancer (2). On the basis of the incidence rates alone, breast cancer is the most common cancer type among women, and prostate is the most common among men. However, lung cancer is the leading killer among cancers in both men and women in the United States, with age-adjusted mortality rates of 46.7 per 100,000 people in men and 31.9 per 100,00 people in women (3, 4). Patients with lung cancer are often diagnosed at an advanced stage, leaving patients with limited options for treatment and a low probability of survival (5, 6).

Although varying by cancer type, the extraordinarily high cost of cancer treatment can significantly negatively affect a patient's life. This effect has come to be called financial toxicity (7). have classified the three main domains contributing to financial toxicity into materialistic, psychologic, and behavioral hardship (8–11). Materialistic hardship arises from skyrocketing out-of-pocket costs and a lowered income due to work impairments. Psychologic hardship could arise due to sacrifice, anxiety about paying current bills or incurring debt, concerns about the future financial burden, and other household expenses. Finally, coping behaviors that the patients are forced to adopt to manage their medical care while experiencing increased expenses and decreased income lead to financial and mental distress (12, 13). Coping behaviors adopted by the patients themselves may lead to financial and mental distress but mostly they are a manifestation of that distress and may lead to poor outcomes. All three domains are intertwined and negatively affect patient's health outcomes.

Costs can be classified into “direct medical costs” and “indirect costs.” The direct medical costs are those associated with services that patients receive, including hospitalizations, surgery, physician visits, radiotherapy, chemotherapy, and immunotherapy (14–16). Insurance payments and patient out-of-pocket costs such as copayments, coinsurance, and deductibles typically measure direct medical costs. Indirect costs of cancer are the monetary losses associated with time spent receiving medical care, time lost from work or other usual activities (morbidity costs), and lost productivity due to premature death (mortality costs). These costs are incurred by patients as well as their caregivers and families. Indirect costs are typically reflected in monetary transactions; however, it is important to recognize that the value of lost time can only be approximated.

The cost of treatment often varies based on the type of cancer, its severity, and the treatment (17, 18). The type of insurance often determines the out-of-pocket costs incurred during treatment, and studies have used out-of-pocket cost to investigate the financial and personal hardships faced by patients with cancer (19, 20). A Canadian study summarized lung cancer financial burden using a formulaic metric which showed that Canadians of younger age and without private insurance were at a greater risk of financial burden (21). An American study showed that older Americans with cancer suffered from higher out-of-pocket costs when compared with those without cancer (22). Individuals living in poverty have been shown to suffer from higher cancer incidence and mortality (23–25). Previous studies have rarely had access to a comprehensive data set that includes patient's clinical, financial, and coping behaviors documented in a single data source. One study utilized a dataset similar to ours but only evaluated the depletion of cancer patient's savings over time (26).

The primary goal of this study is to predict a worsening health outcome by modeling materialistic, psychologic, and behavioral factors. We seek to evaluate key financial factors impacting the health outcomes among patients with cancer who are diagnosed with either breast, lung, prostate, or colon cancer.

## Materials and Methods

The study protocol was approved by the Institutional Review Board at the University of Kansas Medical Center (STUDY00147028). The appropriate review and approval process defined by the Health Retirement Study team was followed to access the restricted data at the University of Michigan (Ann Arbor, MI). The Health and Retirement Study (HRS) was approved by the Institutional Reviewing Board at the University of Michigan and the National Institute on Aging (HUM00061128). All participants filled in the informed consent forms.

To evaluate the financial factors that lead to worsening health outcomes among cancer patients’ observations were drawn from the University of Michigan HRS (restricted dataset). HRS is a national longitudinal study conducted by the Institute for Social Research (ISR) at the University of Michigan with a focus on economic, health, marital, and family status, as well as public and private support systems, of older Americans (27). HRS is a rich source of longitudinal, cross-sectional data for researchers and policymakers who study aging, and surveys approximately 20,000 people 50 years or older in America every two years and includes questions regarding cancer. Funding for the HRS is provided by the National Institute on Aging at the NIH (U01AG009740), with supplemental support from the Social Security Administration.

Participants who had a diagnosis of either breast, lung, prostate, or colon cancer were flagged, and their year of diagnosis served as their baseline from which the 2-year and 4-year postdiagnosis response served as the two follow-up time points. The diagnosis information is restricted data for which we made a formal request to the University of Michigan HRS team for access. Given that new variables were included related to cancer in 2002, our cohort focused on participants who responded from 2002 to 2016. Participants who had a cancer diagnosis in the year 2016 were dropped because we did not have their follow-up responses. This resulted in a dataset of 1,136 patients with cancer across the four cancer types.

**FIGURE 1:**
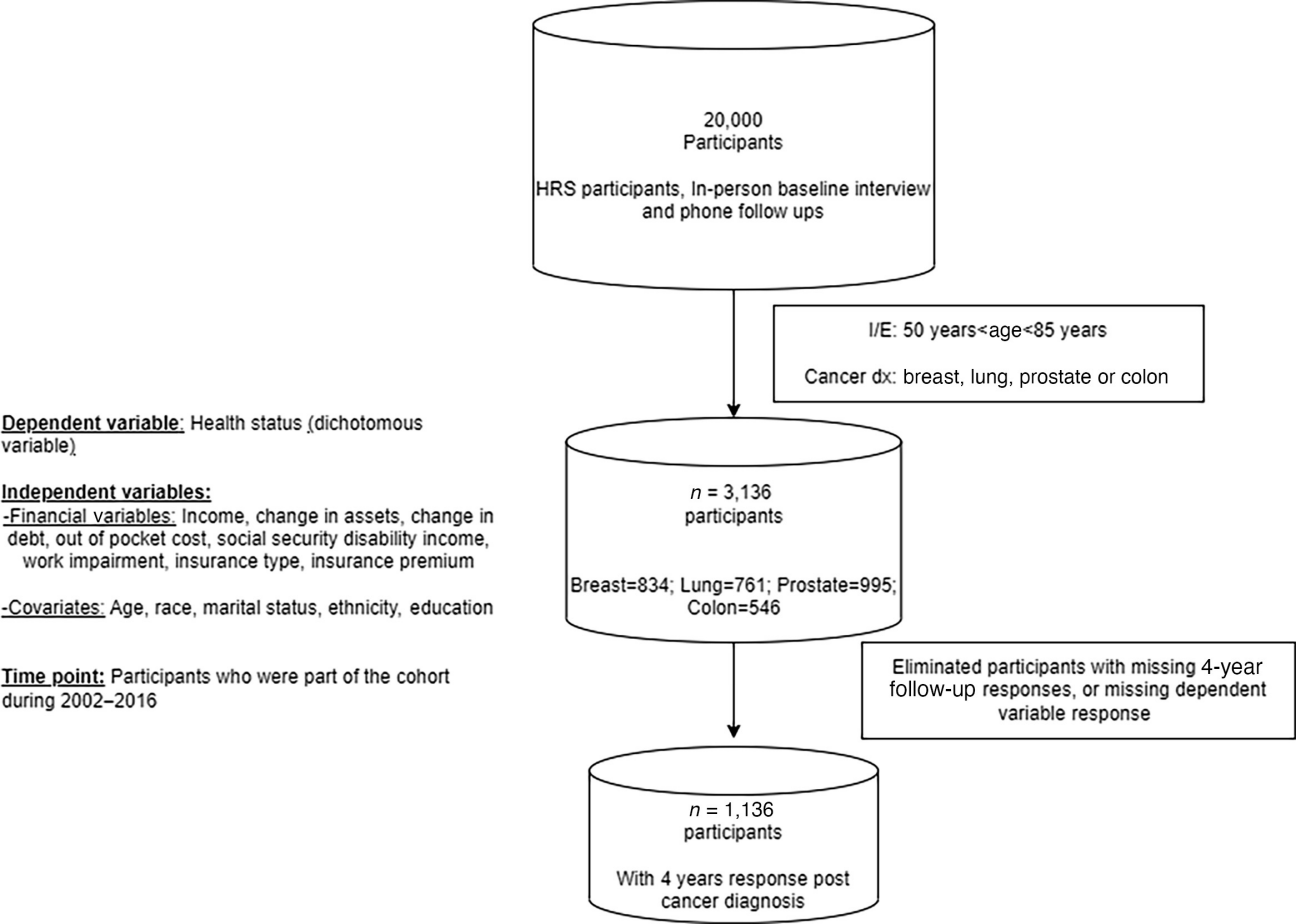
Analytic cases.

The dependent variable of interest from this dataset was overall health status. The independent variables were work impairment, out-of-pocket cost, social security disability income, poverty level, change in assets value, household income, and change in debt value. Smoking status, depression, education level, race, ethnicity, and gender were also used as covariates. Figure 1 below represents how our analytic cases were derived from the HRS dataset.

### Factors

The conceptual framework was developed as many patients with cancer face materialistic hardship, including high out-of-pocket expenses and work impairments; psychologic burden from distress and anxiety caused by the high financial expense and the depletion of savings; and behavioral changes resulting from high cancer treatment costs, which includes both direct and indirect costs will directly impact health outcomes.

### Dependent Variable

For this analysis, we used the variable of overall health status to be the outcome variable of interest. This is a self-reported question documenting health status change from the previous to the current interview. The exact question that is asked during the interview is as follows:

Compared with your health when we talked with you in R's LAST IW MONTH (per Z092), YEAR (per Z093), would you say that your health is better now, about the same, or worse?

This variable has responses of “much better”, “somewhat better”, “same”, “somewhat worse”, and “much worse”. This variable was dichotomized into worsening health (1) for individuals who answered, “somewhat worse”, and “much worse” and 0 for the other responses.

### Independent Variables

The out-of-pocket cost was recorded as a dollar amount. To make these amounts comparable across participants, we scaled this variable and derived a proxy that is out-of-pocket cost per $10,000 spent on medical care.

The variables of work impairment and poverty threshold were dichotomous “yes”/“no” response variables. The exact question that was posed to the patients was – “Do you have any impairment or health problem that limits the kind or amount of paid work you can do?” The poverty threshold was a computed dichotomous variable based on gross income above/below the federal poverty level (>100% FPL) for the year of the survey.

Social security disability income, change in assets value, household income, and change in debt value were continuous dollar amounts that were adjusted similarly to out-of-pocket costs, to compare across every patient addressing some of the extreme outliers that exist within the dataset.

### Covariates

Race had three response options, and “White” acted as the reference category for “African American” and “Other.” The response distribution and previous literature suggested that patients of race white were different from the other race groups. Ethnicity was a dichotomous variable as either “Hispanic” or “non-Hispanic”. was a dichotomous variable of “Male” or “Female”.

Education was a variable that had multiple options including high school, GED, Bachelors, Masters, and less than high school. We derived a dichotomous variable in which “1” was assigned to participants who possessed a college degree or higher and “0” was assigned to participants who had a degree less than college.

### Primary and Secondary Analysis

The primary endpoint for this study is self-reported health status change at 4-year time point post cancer diagnosis. Health status change was captured at every interview; participants were asked whether their health had deteriorated, remained the same, or gotten better relative to their previous response. The secondary analysis consisted of subgroup analyses of predicting worsening health outcome by individual cancer type. As an exploratory analysis, we independently conducted a covariate analysis using logistic regression with the moderator variables of age, race, gender, ethnicity, education and marital status to ensure robustness of our findings.

### Statistical Analysis

To describe the data, we first ran summary statistics. These included means and SDs for continuous variables, and frequencies and percentages for categorical. Variables included income, change in assets, change in debt, out of pocket cost, social security disability income, work impairment, age, race, marital status, ethnicity, education, insurance type and insurance premium. models were assessed for normality through visualization. A stepwise logistic regression model was used to identify the significant financial factors that predict a worsening health status among patients with cancer. Forward selection was used to identify the top financial risk factors influencing worsening health status, and selection was made to include 2, 3, 4, and 5 terms. Models were compared by their Akaike information criterion (AIC), C statistic, and percent concordant while maintaining parsimony and all factors in the model being statistically significant (*P* ≤ .05). Adjusted ORs and 95% confidence intervals (CI) are reported for the best two-, three-, four-, five-, and six-factor models. On the basis of the forward selection models, factors identified were reported as ORs, 95% CIs, and *P* values.

In addition, logistic regression was conducted to examine the interaction between the factors in the model. Three-way and two-way interactions were examined to ensure there were no significant interactions among the variables in the best-fit model.



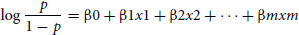



Where log(probability of worse health outcome/1-probability of worse health outcome) = constant + x1 work impairment + x2 out of pocket cost + x3 social security disability income +x4 race + x5 education + x6 poverty level+ x6 change in assets + x7 household income + x8 change in debt + x9 gender + x10 smoking status + x11 ethnicity.

For the secondary analysis, stepwise regression was used again but on smaller data subsets constructed using cancer types of lung, breast, prostate, and colon. Significant predictor variables were then compared across the cancer subset models.

For the exploratory analysis, a logistic model was used with individual moderator variables, and as a sensitivity analysis a comprehensive model with all the moderator variables was used to verify any potential differences in OR.

### Data Availability

The data used to conduct this research was a restricted data set provided by the University of Michigan Health Retirement Study team (approved under application number # RDA:2021-034). Data can be accessed after appropriate approval is obtained (for more information, please follow the link: https://hrs.isr.umich.edu/data-products/restricted-data/available-products/9691). The data product utilized for our study is labeled *cancer sites*, under the product type Health Care Information.

## Results

Table 1 demonstrates participant characteristics. 1,136 participants were included in the main analysis for the study. These participants had a documented response with the cancer type and the diagnosis year, and the immediate follow-up response, which was 2 years and 4 years out from their cancer diagnosis year. The gender distribution was 51.58% female and 48.42% of male. The race of respondents was predominately White (77.11%) followed by 19.54% African American and 3.35% other.

**TABLE 1:** Participant characteristics

	*N* (%)			
Gender				
Female	586 (51.58%)			
Male	550 (48.42%)			
Race				
White	876 (77.11%)			
African American	222 (19.54%)			
Other	38 (3.35%)			
Hispanic				
Not Hispanic	1059 (93.22%)			
Hispanic	77 (6.78%)			
Education				
Less than a college degree	811 (71.39%)			
College degree or above	325 (28.61%)			
Ever smoked				
No	525 (46.21%)			
Yes	611 (53.79%)			
Drinking status				
No	584 (51.41%)			
Yes	552 (48.59%)			
Poverty level (>100% FPL)				
Above poverty level	924 (81.34%)			
Below poverty level	101 (8.89%)			
Unknown	111 (9.77%)			
Work impairment				
No	749 (65.93%)			
Yes	387 (34.07%)			
Insurance type				
Public (Medicaid/Medicare)	170 (14.96%)			
Private	966 (85.04%)			
	**Mean**	**SD**	**Minimum**	**Maximum**
Out-of-pocket cost per year	$3,816.42	$8,921.22	0	$170,940
Income per year	$13,190.05	$47,717.61	0	$607,421.9
Social Security Income or Disability Income per year	$726.80	$3,350.36	0	$41,616
Change in debt over 2 years	$3,579.24	$15,611.6	0	$300,000
Change in assets over 2 years	−$46,801.98	$1,341,849	−$38,100,000	$8,354,000
Premium for public insurance	$61.88	$85.58	0	$366
Premium for private insurance	$215.88	$258.22	0	$2,100

Table 2 below demonstrates the best-fit logistic model to identify the associated financial factors that jointly predicted the worsening health outcome among the cancer patients, overall.

**TABLE 2:** The best-fit model – evaluating the financial factors across four cancer type patients (breast, lung, prostate, and colon)

	Best 2-factor model^a^		Best 3-factor model		Best 4-factor model		Best 5-factor model	
Variable	OR (95% CI)	*P* ^b^	OR (95% CI)	*P* ^b^	OR (95% CI)	*P* ^b^	OR (95% CI)	*P* ^b^
Work impairment	3.54 (2.66–4.71)	<0.0001	3.58 (2.69–4.76)	<0.0001	3.60 (2.70–4.80)	<0.0001	3.71 (2.77–4.98)	<0.0001
Out-of-pocket cost per $10,000 spent	1.19 (1.02–1.40)	0.025	1.19 (1.09–1.39)	0.028	1.19 (1.09–1.39)	0.0285	1.19 (1.02–1.40)	0.026
Change in assets per $100,000			1.02 (0.99–1.04)	0.118	1.02 (0.99–1.04)	0.118	1.02 (0.99–1.04)	0.113
Change in debt per $20,000 incurred debt					0.90 (0.72–1.12)	0.367	0.90 (0.72–1.13)	0.383
Social Security Income							0.98 (0.94–1.02)	0.355
Model fit statistics								
AIC intercept and covariates	1,146.93		1,145.83		1,146.89		1,145.99	
Percent concordant	65.6		66.6		67.1		67	
C-statistics	0.659		0.666		0.672		0.67	

^a^The two-factor model had the best-fit statistics while maintaining parsimony and statistical significance of factors in the model.

^b^ *P* values were calculated for each factor using the two-sided Wald tests. Noting that these models include covariates such as age, gender, education, race, marital status, and ethnicity.

The two-factor model, where work impairment and out-of-pocket cost per $10,000 incurred, predict worsening health outcomes with an AIC intercept and covariates of 1146.93, a percent concordance of 65.6, and a C-statistic of 0.659. The two-factor model is the best fit in this case as adding additional factors did not yield additional explanatory power from the model fit statistics. With the three-factor model as we note there is a decrease in AIC intercept and the covariate. The percent concordance increases by 1 percent with a minor increase of 0.007 in c-statistics compare to two-factor model.

Table 3 demonstrates the results of logistic regression examining the impact of the worse health outcomes due to work impairment, out-of-pocket costs, and their interaction. The nonsignificant *P* value of the interaction term implies that both variable effects are operating independently.

**TABLE 3:** Logistic regression of full model of main effects

	Full model	
Variable	OR (95% CI)	*P* ^a^
Work impairment	3.17 (2.31–4.36)	<0.0001
Out-of-pocket cost per $10,000 spent	1.07 (0.87–1.31)	0.481
Work Impairment × Out-of-pocket cost per $10,000 spent	1.30 (0.92–1.84)	0.125

^a^ *P* values were calculated for each factor using two-sided Wald tests.

In the secondary subset analysis, “work impairment” remained the best predictor across all cancer types for the two-factor model, and “Out of pocket cost per $10,000 spent” remained the next-best for all cancers. No clear pattern existed across cancer types in the three-factor and above models.

The ORs from the covariate analysis for gender with the reference group set as a female in comparison with male suggests that both work impairment (2.99; 95% CI, 2.40–3.71; *P* < 0.001) and out-of-pocket costs (1.26; 95% CI, 1.11–1.44; *P* < 0.001) were significant which is similar to the primary analysis. For race with white as the reference group, both work impairment (3.01; 95% CI, 2.42–3.74; *P* < 0.001) and out-of-pocket cost (1.27; 95% CI, 1.12–1.45; *P* < 0.001) were significant financial factors in predicting worsening health status. For ethnicity with non-Hispanic as the reference group, the variables of work impairment (3.00; 95% CI, 2.418–3.74; *P* < 0.001), out-of-pocket cost (1.27; 95% CI, 1.12–1.45; *P* < 0.001) and poverty level (1.49; 95% CI, 1.03–2.16; *P* = 0.034) were significant. Among the younger patients with cancer in the dataset (defined as patients who were between the age of 50 to 64 years), work impairment (3.06; 95% CI, 2.46–3.81; *P* < 0.001), and out-of-pocket cost (1.24; 95% CI, 1.19–1.45; *P* < 0.001) were significant predictors when compared with older patients (65 years and above). In the context of education, patients who did not possess a college degree showed work impairment (2.95; 95% CI, 2.37–3.68; *P* < 0.001) and out-of-pocket cost (1.28; 95% CI, 1.12–1.46; *P* < 0.001) as a significant predictor for worsening health status. For marital status, with married being the reference group work impairment (3.00; 95% CI, 2.41–3.73; *P* < 0.001), out-of-pocket cost (1.27; 95% CI, 1.12–1.45; *P* < 0.001), and poverty status (1.47; 95% CI, 1.02–2.13; *P* = 0.039) were significant predictors.

## Discussion

Our primary aim was to identify the financial factors that significantly contribute toward predicting worsening health outcomes among the most common cancer patients, that is, breast, lung, prostate, and colon. To this extent, our forward stepwise regression model based on the fit statistics suggests that the key financial factors that significantly worsen cancer patient's health outcomes are work impairments and out-of-pocket costs. It is important to note the financial toxicity factors that added relatively little to predicting worsening health outcomes – for example, poverty, household income, social security insurance income, change in debt, and change in assets.

We found that among all the socioeconomic factors analyzed, the most influential on cancer patients’ financial burden were work impairment and out-of-pocket costs. The majority of the out-of-pocket cost has to do with cancer drug pricing; this was assessed on the basis of a subsequent question that was administered as part of the survey. A lack of policies to govern and moderate drug prices is taking a major toll on patients with cancer. Similarly, some patients who are diagnosed with cancer need to quit their jobs. Some patients are forced to take a longer break from work or even completely change their careers. This can cause the loss of significant psychologic and financial benefits for patients (28). Professional derailment not only impacts their ability to earn money but also has additional long-term impacts, such as loss of confidence and a negative impact on their postrecovery outlook.

Losing a job or even opting for short-term or long-term disability means reduced income and loss of employer benefits. Typically, if the patient with cancer is the primary breadwinner, then a diagnosis has a cascading impact on the entire household. Four years after diagnosis, patients with breast cancer treated with chemotherapy have 1.42 times the odds of nonemployment compared with those without cancer (28).

We have noted that 60% of workers across the United States are not entitled to any form of sick leave or time off. Many workers who have a higher probability of being exposed to carcinogens are more prone to lack of Family and Medical Leave Act (FMLA) and sick leave benefits (23). A policy that guarantees sick leave or time off could be developed, especially for workers who encounter cancer, as cancer treatment journey is long (22). A study investigating head and neck cancer found that a supportive work environment was positively associated with patients return to work (29).

Effective strategies to mitigate the cost of expensive cancer drugs have not been developed. Limited literature discusses and highlights how pharmaceutical companies have frequently claimed that high launch prices are due to their spending on research and development, which has led to skyrocketing prices (30). It is time for policymakers and regulatory bodies to step in so that cancer drugs also follow the regulations and have an upper ceiling, and the prices of generic drugs must taper off over one to two years after their launch or once they have recouped their research and development cost. Regulatory bodies must also ensure only limited generic drugs can be approved to keep the pharmaceutical companies from jeopardizing their patents and ensure the manufacturing of approved drugs is manufactured in the right quantity to meet the demand. Policies must be implemented where pharmaceutical companies work together to develop cancer drugs that could be more effective in producing cutting-edge cancer therapies rather than duplicating efforts and wasting resources, which in turn increases drug prices. Pharmaceutical companies also have started investigating a topic called clinical trial optimization, where the time to evaluate the efficacy of a cancer drug could be reduced to 3 to 5 years instead of a typically 8- to 10-year period.

### Limitations

It is difficult to generalize results across all cancer types. Each cancer type is different in the nature of the disease, the available treatment options, and the way how it affects a patient's overall health. The lack of employer benefits that patients had during and posttreatment is unknown. Employer benefits could be an influential factor in the financial toxicity among patients with cancer.

The HRS dataset only includes individuals over the age of 50 and as such this limits our findings to only this age group. We cannot make findings about how cancer diagnosis impacts younger individuals using this dataset. The age group of 50 and older represents most of the patients with cancer, but it does not represent most of the workforce., the HRS dataset is a 2-year retrospective survey response which is subjected to recall bias. Individuals may recall their situation drastically better or worse than it was two years ago (31). This is an inherent issue with retrospective data collection.

## Conclusion

Work impairment and out-of-pocket costs were the two financial factors most robustly associated with worsening health outcomes among patients with cancer. The subgroup analysis by cancer type showed a similar pattern. Past research has not evaluated work impairment as one of the financial factors contributing to financial burden among cancer patients, which is one of the key discoveries of our study. Work impairment immensely impacts the ability of cancer patients to continue to earn money and have access to employer-sponsored benefits.

Over the past decade, the topic of financial toxicity has gained significant attention, and policymakers anticipated the Affordable Care Act (ACA) would address the financial burden along with the access to care issues among patients with cancer. A continued emphasis on prevention and early detection through modern screening techniques would help by allowing for early intervention (18,32,33). Although access to care and cancer screening has dramatically improved, the cost of treatment and disparities still widely exists, leading to poor financial and health outcomes among patients with cancer.
